# Impact of skeletal muscle mass evaluating methods on severity of metabolic associated fatty liver disease in non-elderly adults

**DOI:** 10.1017/S0007114523000399

**Published:** 2023-10-28

**Authors:** Ting Zhou, Junzhao Ye, Yansong Lin, Wei Wang, Shiting Feng, Shuyu Zhuo, Bihui Zhong

**Affiliations:** 1 Department of Gastroenterology, The First Affiliated Hospital, Sun Yat-sen University, No. 58 Zhongshan II Road, Yuexiu District, Guangzhou 510080, People’s Republic of China; 2 Department of Medical Ultrasonics, Institute of Diagnostic and Interventional Ultrasound, The First Affiliated Hospital, Sun Yat-sen University, No. 58 Zhongshan II Road, Yuexiu District, Guangzhou, Guangdong 510080, People’s Republic of China; 3 Department of Radiology, The First Affiliated Hospital, Sun Yat-sen University, No. 58 Zhongshan II Road, Yuexiu District, Guangzhou, Guangdong 510080, People’s Republic of China; 4 Department of Nutrition, The First Affiliated Hospital, Sun Yat-sen University, No. 58 Zhongshan II Road, Yuexiu District, Guangzhou, Guangdong 510080, People’s Republic of China

**Keywords:** Metabolic associated fatty liver disease, Low muscle mass, Steatosis, Liver fibrosis, Insulin resistance

## Abstract

The study aimed to explore the relationships of skeletal muscle mass with disease severity in metabolic-associated fatty liver disease (MAFLD) patients with different methods. Consecutive subjects undergoing bioelectrical impedance analysis were included. The steatosis grade and liver fibrosis were evaluated by MRI-derived proton density fat fraction and two-dimensional shear wave elastography. The appendicular skeletal muscle mass (ASM) was adjusted by height^2^ (ASM/H^2^), weight (ASM/W) and BMI (ASM/BMI). Overall, 2223 subjects (50·5 %, MAFLD; 46·9 %, male) were included, with the mean age 37·4 ± 10·6 years. In multivariate logistic regression analysis, the subjects with the lowest quartile (Q1) of ASM/W or ASM/BMI had higher risk ratios for MAFLD (OR (95 % CI) in male: 2·57 (1·35, 4·89), 2·11(1·22, 3·64); in female: 4·85 (2·33, 10·01), 4·81 (2·52, 9·16), all *P* < 0·05, all for Q1 *v*. Q4). The MAFLD patients with lower quartiles of ASM/W had the higher risk OR for insulin resistance (IR), both in male and female (2·14 (1·16, 3·97), 4·26 (1·29, 14·02) for Q4 *v*. Q1, both *P* < 0·05). While the significant OR were not observed when ASM/H^2^ and ASM/BMI were used. There were significant dose-dependent associations between decreased ASM/W as well as ASM/BMI and moderate–severe steatosis (2·85(1·54, 5·29), 1·90(1·09, 3·31), both *P* < 0·05) in male MAFLD patients. In conclusion, ASM/W is superior to ASM/H^2^ and ASM/BMI in predicting the degree of MAFLD. A lower ASM/W is associated with IR and moderate–severe steatosis in non-elderly male MAFLD.

Metabolic-associated fatty liver disease (MAFLD), also referred to as non-alcoholic fatty liver disease (NAFLD), is characterised by diffused fat infiltration in hepatocytes as well as presentations of metabolic dysregulation^(1,2)^. Based on the current prevalence of 26·9 % in China, it is predicted that MAFLD will affect more than 3 billion people by 2030^(3,4)^. This growing disease burden poses a considerable threat to public health, because MAFLD may not only progresses to steatohepatitis, cirrhosis or hepatocellular carcinoma but also confers high risks of various metabolic disorders, including obesity, insulin resistance (IR), dyslipidemia, hyperglycaemia and CVD^(5)^. Therefore, it is imperative to identify key determinants of metabolic co-morbidities to improve prognosis^(6)^.

Sarcopenia is defined as progressive loss of muscle mass, muscle strength and physical activity function with ageing^(7)^. Since muscle is the major organ involved in energy expenditure through motility and physical activities, there is growing interest in positive associations between muscle mass and metabolic abnormalities such as IR^(8)^, diabetes^(9)^ and metabolic syndrome^(10)^. Moreover, MAFLD and sarcopenia share several common pathophysiological processes and receive much clinical attention^(11)^. Most reports linking the muscle mass and MAFLD have been conducted in general populations by bioelectrical impedance analysis (BIA), revealing an association between the decreased muscle mass and overrepresented prevalence of significant hepatic steatosis and fibrosis^(12–14)^. However, the assessment methods for muscle mass in these studies varied, presented by appendicular skeletal muscle mass (ASM) adjusted by body weight, height^2^ or BMI on the basis of body composition analysis with bioelectrical impedance measurements. In a previous study collecting data for 11 065 subjects in the Third National Health and Nutrition Examination Survey, which utilised the skeletal muscle index derived from bioelectrical impedance measurements to screen for sarcopenia, 28 % of healthy individuals were categorised as having sarcopenia, possibly causing biased estimation and therefore misinterpretation of its association with NAFLD^(15)^. Another study including 1343 healthy individuals undergoing check-up found that sarcopenia defined merely using height^2^ instead of body weight adjustment showed a positive relationship with NAFLD-related metabolic abnormalities^(16)^. Nevertheless, whether MAFLD as well as liver condition is closely related to sarcopenia determined by different methods remains uncertain. Hence, illustrating the impact of different assessments of muscle mass on their relationships with MAFLD is warranted. Muscle mass peak in young adulthood and after a plateau start decreasing gradually^(17)^. However, previous studies explored the association of sarcopenia and MAFLD in elderly people^(18–20)^ or adults aged ≥ 18 years^(12,16,21,22)^, the investigation on which was lack in non-elderly population. The impact of muscle mass loss on non-elderly MAFLD is not clear and need to be explored.

Therefore, our study aimed to explore the characteristics of muscle mass in non-elderly patients with MAFLD using different screening strategies including ASM adjusted by height^2^, weight, and BMI, furthermore to analyse the relationship of which with IR, steatosis grades determined by MRI-derived proton density fat fraction (MRI-PDFF), and hepatic fibrosis determined by two-dimensional shear wave elastography (2D-SWE).

## Materials & methods

### Study design and subjects

This was a cross-sectional study, consecutively enrolled subjects who underwent BIA in the First Affiliated Hospital, Sun Yat-sen University, between May 2017 and July 2022. This study was conducted according to the guidelines laid down in the Declaration of Helsinki, and all procedures involving patients were approved by the ethics committee of the First Affiliated Hospital, Sun Yat-sen University (Approval number: (2014) 112). And the informed consent was obtained from all participants.

The eligibility criteria for the study were as follows: (a) 18–60 years old, (b) underwent abdominal ultrasonography, and (c) underwent BIA. The exclusion criteria included (a) hepatocellular carcinoma, (b) autoimmune liver disease, Wilson’s disease, drug-induced liver disease, (c) heavy alcohol consumption (> 140 g/week for males or > 70 g/week for females), (d) taking medications that could induce steatosis or affect body weight (steroid, tamoxifen, etc), (e) diagnosis of extrahepatic malignancies within the past year, and (f) pregnancy.

### Clinical and laboratory indices

Demographic information was collected, and anthropometric indices including weight, height, waist and hip circumference were measured. Height and weight were measured to the nearest 0·1 cm and 10 g using a height and weight measuring instrument (Omron NHN-219). Subjects were required to stand on the instrument with lightweight clothes, their shoes removed and their arms hanging freely. Waist circumference was measured at the midpoint between the lower margin of the rib cage and the top of iliac crest using a non-elastic measuring tape, and hip circumference was measured at the widest point between the hip and buttock. The waist:hip ratio was calculated as the waist circumference (cm) divided by hip circumference (cm), and the BMI was calculated as weight (kg) divided by height^2^ (m^2^). Blood samples were drawn after patients had fasted for 8 h to measure the following indices: alanine aminotransferase, aspartate aminotransferase, *γ*-glutamyl transpeptidase, alkaline phosphatase, total cholesterol, TAG, LDL-cholesterol, HDL-cholesterol, fasting serum glucose, fasting insulin, glycated haemoglobin A1c (HbA1c) and uric acid. The homeostasis model assessment of insulin resistance (HOMA-IR) was calculated as follows: HOMA-IR = fasting serum glucose (mmol/l) × fasting insulin (μU/ml)/22·5^(23)^.

### Severity of metabolic-associated fatty liver disease assessment

The diagnosis of MAFLD was based on criteria approved by an international expert panel^(24)^. Liver fat content measurements was utilised to assess the severity of steatosis for all MAFLD patients, which was obtained by MRI-PDFF with a 3.0-Tesla MRI scanner (Siemens 3.0T MAGNETOM Verio). MRI-PDFF was performed by two trained radiologists blinded to the aim of this study. The scanning protocol and imaging parameters were the same as described in our previous published study^(25)^. The steatosis grade was graded as mild (5–10 %) and moderate–severe (≥ 10 %), which were validated in previous study^(26)^.The liver fibrosis was evaluated by liver stiffness measurement conducted by 2D-SWE (Aix-en-Provence, France) by two physicians with over 3 years of experience blinded to the clinical information of the study. The subjects were determined significant liver fibrosis when liver stiffness measurement ≥ 7·1 kPa^(27)^. IR was defined by the HOMA-IR ≥ 2·5^(13)^.

### Skeletal muscle mass assessment

The BIA was performed by a segmental multifrequency bio-resistance body composition analyser according to the manufacturer’s instructions (TANITA, MC-980MA). Before BIA measurement, the subjects have fasted overnight (at least 8 h) and were required to avoid vigorous exercise. The patients were instructed to stand on the evenly on the electrodes under the toes and heels and hold a handle in each hand. All participants spread apart their limbs to ensure that their arms did not touch the trunk and the thighs were not in contact, remaining motionless for 40 s during the measurement. The impedance for each segments including four limbs and the trunk were provided, and the device estimated skeletal muscle mass by calculation from regression equations developed by Yamada et al.^(28)^ The ASM was calculated by the sum of the lean muscle mass of the upper and lower limbs, which were measured directly by BIA. The appendicular skeletal muscle index (ASMI) was adjusted in three different ways: ASMI (kg/m^2^) = ASM (kg)/height^2^ (m^2^) (ASM/H^2^), ASMI (%) = ASM (kg)/weight (kg) × 100 % (ASM/W) and ASMI = ASM (kg)/BMI (kg/m^2^). The cut-off values for low muscle mass (LMM) were defined by ASM/H^2^ (< 7·0 kg/m^2^ for males, < 5·7 kg/m^2^ for females)^(29)^, ASM/W (< 29·0 % for males, < 22·9 % for females)^(14)^ and ASM/BMI (< 0·789 for males, < 0·512 for females)^(30)^. In this study, the prevalence of LMM was evaluated by five ways: ASM/H^2^, ASM/W, ASM/BMI, ASM/W* and S_0_ (at least one of four mentioned above positive, ASM/H^2^ or ASM/W or ASM/BMI or ASM/W*). The cut-offs for ASM/W* were < 29·5 % for males or < 23·5 % for females and were calculated according to the Asian Working Group for Sarcopenia^(31)^, which recommends using 2 sd below the mean muscle mass of young reference group as the cut-off value determination. The cut-offs of LMM for men and women were based on two sd below the sex-specific mean of a younger healthy population (18–39 years) from this study. Handgrip strength was measured using a Camry EH101 electronic hand dynamometer^(32)^, and the participants were asked to hold the dynamometer (to place their first fingers over the outer handle of the dynamometer and the others over the inner handle) in the hand with their elbows flexed at 90° and other parts of body still. During the test, the subjects were asked to squeeze the dynamometer with their maximum strength for approximately 5 s. The tests were conducted three times (with a break of at least 1 min between times) to obtain the average values as the representative results.

### Histological evaluation

In this study, liver biopsy was conducted by 18G Temno needles under the ultrasound guidance to get two samples at least 15 mm in length in the right hepatic lobe for each patient. All liver specimens were assessed independently by two fixed pathological experts with over 10-year experience, who were blinded to the study data. The third pathologist participated in the discussion to achieve a final consensus if there were inconsistencies in the assessments. According to histological analysis, mild, moderate and severe steatosis was defined by the presence of steatosis in 5–33·3 % (S1), 33·4–66·6 % (S2) and ≥ 66·7 % (S3) of hepatocytes. Fibrosis was graded using the Kleiner fibrosis score^(33,34)^. Absence and presence of fibrosis were defined as F0 and F1–F4, respectively.

## Statistical analysis

Continuous variables were reported as means ± the sd, and categorical variables as numbers (n) and percentages (%). The statistical significance of differences between groups was evaluated using the independent *t* test, the Mann–Whitney *U* test, non-parametric rank sum test for continuous variables and the *χ*^2^ test for categorical variables. The multivariate logistic regression analyses were applied to determine the association between ASMI quartiles and MAFLD, IR, steatosis grade and liver fibrosis, the highest quartile used as the reference group. *P* values < 0·05 were considered significant. All statistical analyses were performed using SPSS Statistics version 25.0 software (IBM) and GraphPad Prism 8 (Inc.).

## Results

### Baseline characteristics of the study population

**Fig. 1.**
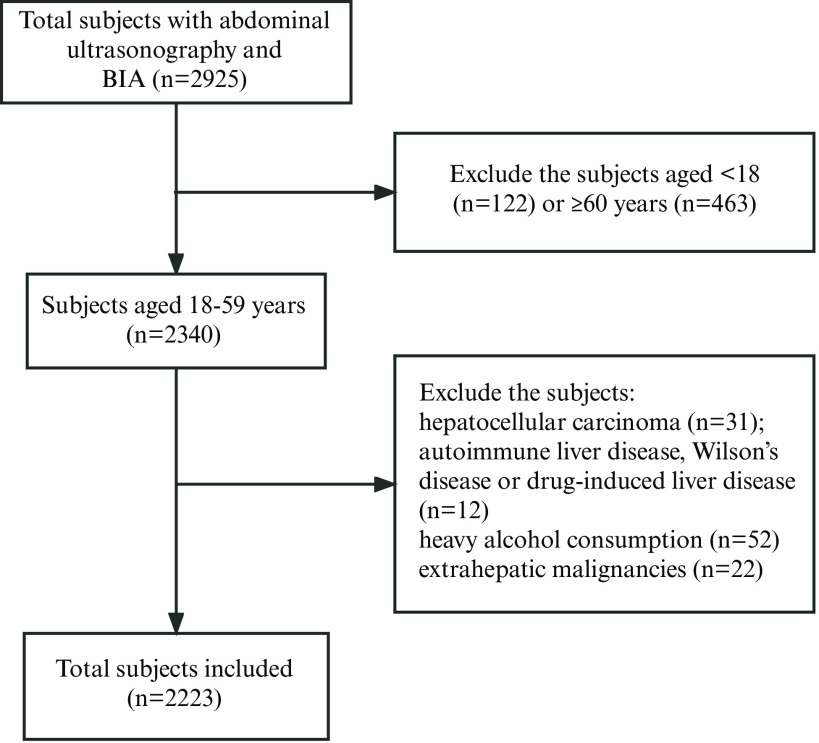
Flow diagram of subject inclusion and exclusion. BIA, bioelectrical impedance analysis.

Overall, 2223 subjects (46·9 %, male) were eligible and included in the study (Fig. 1), consisting of 1123 (50·5 %) subjects with MAFLD. Demographics, anthropometric, liver biochemistry, metabolic indices and body component characteristics grouped by MAFLD diagnosis and sex are presented in Table 1. MAFLD patients had higher levels of weight, BMI, waist circumference, alanine aminotransferase, aspartate aminotransferase, total cholesterol, TAG, LDL-cholesterol, and fasting serum glucose and lower HDL-cholesterol levels compared with those without MAFLD. Higher ASMI adjusted by height^2^ but lower ASMI adjusted by weight or BMI were found in both males and females with MAFLD than in those without MAFLD (Table 1). The prevalence of LMM was higher in non-MAFLD than in MAFLD, both for males and females, when LMM was defined by ASM/H^2^ and S_0_. However, the prevalence was higher in MAFLD than non-MAFLD when LMM was identified merely by ASM/W and ASM/W* (Table 1). There was no difference between MAFLD and non-MAFLD in handgrip strength both in male and female.

**Table 1.** Comparison of the anthropometry, metabolic and body composition characteristics between the MAFLD and non-MAFLD patients

	Men (*n* 1042)					Women (*n* 1181)				
	MAFLD		Non-MAFLD			MAFLD		Non-MAFLD		
	(*n* 659, 63·2 %)		(*n* 383, 36·8 %)			(*n* 464, 39·2 %)		(*n* 717, 60·7 %)		
Characteristics	Mean	sd	Mean	sd	*P*	Mean	sd	Mean	sd	*P*
Demographics										
Age (years)	37·1	10·5	38·9	11·1	0·11	38·9	10·8	38·1	11·1	0·18
Anthropometry										
Weight (kg)	83·0	18·0	67·6	13·9	< 0·001	73·4	12·7	57·2	10·3	< 0·001
BMI (kg/m^2^)	28·9	5·8	23·1	4·2	< 0·001	28·9	4·2	22·7	3·7	< 0·001
Waist circumference (cm)	97·1	11·2	83·9	10·9	< 0·001	94·0	10·1	79·7	10·2	< 0·001
Waist:hip ratio	0·87	0·07	0·87	0·06	0·680	0·88	0·11	0·85	0·06	< 0·001
	OR	95 % CI	OR	95 % CI		OR	95 % CI	OR	95 % CI	
Liver biochemistry										
Alanine aminotransferase (U/L)	42	26, 69	21	15, 33	< 0·001	24	15, 38	12	15, 20	< 0·001
Aspartate aminotransferase (U/L)	30	23, 42	23	18, 28	< 0·001	22	18, 31	19	17, 22	< 0·001
* γ*-Glutamyl transpeptidase (U/L)	39	28, 65	26	20, 36	< 0·001	27	22, 38	18	13, 27	< 0·001
Alkaline phosphatase (U/L)	75	66, 88	70	59, 80	< 0·001	70	60, 80	65	55, 74	< 0·001
	Mean	sd	Mean	sd		Mean	sd	Mean	sd	
Metabolic characteristics										
Total cholesterol (mmol/l)	5·3	1·1	4·9	1·0	< 0·001	5·6	1·5	4·9	1·0	< 0·001
TAG (mmol/l)										
OR	1·8		1·1		< 0·001	1·5		0·9		< 0·001
95 % CI	1·2, 2·5		0·8, 1·7			1·1, 2·2		0·6, 1·2		
HDL-cholesterol (mmol/l)	1·1	0·6	1·3	0·4	< 0·001	1·3	0·4	1·5	0·3	< 0·001
LDL-cholesterol (mmol/l)	3·3	0·8	3·1	0·8	< 0·001	3·5	1·2	3·0	0·8	< 0·001
Fasting serum glucose (mmol/l)	5·5	1·6	5·2	1·4	0·003	5·6	1·6	5·0	0·7	< 0·001
HOMA-IR										
OR	2·4		1·3		< 0·001	2·1		1·4		< 0·001
95 % CI	1·6, 3·7		0·9, 2·1			1·5, 3·5		0·9, 2·2		
Diabetes										
*n*	111		31		< 0·001	87		31		< 0·001
%	16·8		8·1			19·4		4·3		
HbA1c (%)	6·0	1·3	5·5	0·7	< 0·001	7·0	1·2	5·7	0·3	< 0·001
Diabetes duration (months)†										
OR	25		8		0·004	31		5		< 0·001
95 % CI	6, 62		4, 12			8, 40		3, 9		
Antidiabetic drugs†										
*n*	42		7		0·11	30		12		0·67
%	37·8		22·6			34·5		38·7		
Uric acid (μmol/l)	444·3	107·7	394·2	89·5	< 0·001	346	93	300	79	< 0·001
Handgrip strength (kg)	37·4	8·5	36·9	10·4	0·69	22·8	5·8	23·1	5·9	0·81
Body composition										
ASM (kg)	28·2	5·7	24·4	4·8	< 0·001	19·5	2·8	17·3	2·7	< 0·001
ASM/H^2^ (kg/m^2^)	9·5	1·6	8·4	1·4	< 0·001	7·7	0·9	6·8	0·9	< 0·001
ASM/W (%)	33·6	2·9	36·5	0·4	< 0·001	26·6	2·5	30·3	3·8	< 0·001
ASM/BMI (m^2^)	0·99	0·14	1·06	0·2	< 0·001	0·69	0·23	0·77	0·12	< 0·001
LMM										
	*n*	%	*n*	%		*n*	%	*n*	%	
ASM/H^2^	7	1·1	37	9·7	< 0·001	3	0·6	44	6·1	< 0·001
ASM/W	17	2·6	2	0·5	0·031	20	4·3	5	0·7	< 0·001
ASM/BMI	9	1·4	10	2·6	0·15	4	0·9	1	0·1	0·16
ASM/W*	20	3·0	5	1·3	0·08	26	5·6	8	1·1	< 0·001
S_0_	29	4·4	45	11·7	< 0·001	21	4·5	48	6·7	0·12

MAFLD, metabolic-associated fatty liver disease; HOMA-IR, homeostasis model assessment of insulin resistance; HbA1c, glycated haemoglobin A1c; ASM, appendicular skeletal mass; ASM/H^2^, ASM/height^2^; ASM/W, ASM/weight; LMM, LMM, low muscle mass.

* ASM/W* = ASM/W × 100 %, beyond 2 sd below the sex-specific mean for healthy young adults in this study; S_0_, satisfied ASM/H^2^ or ASM/W or ASM/BMI or ASM/W*.

† Diabetes duration and antidiabetic drugs were available in the patients diagnosed by diabetes.

### Impact of assessment methods on low muscle mass prevalence

**Fig. 2.**
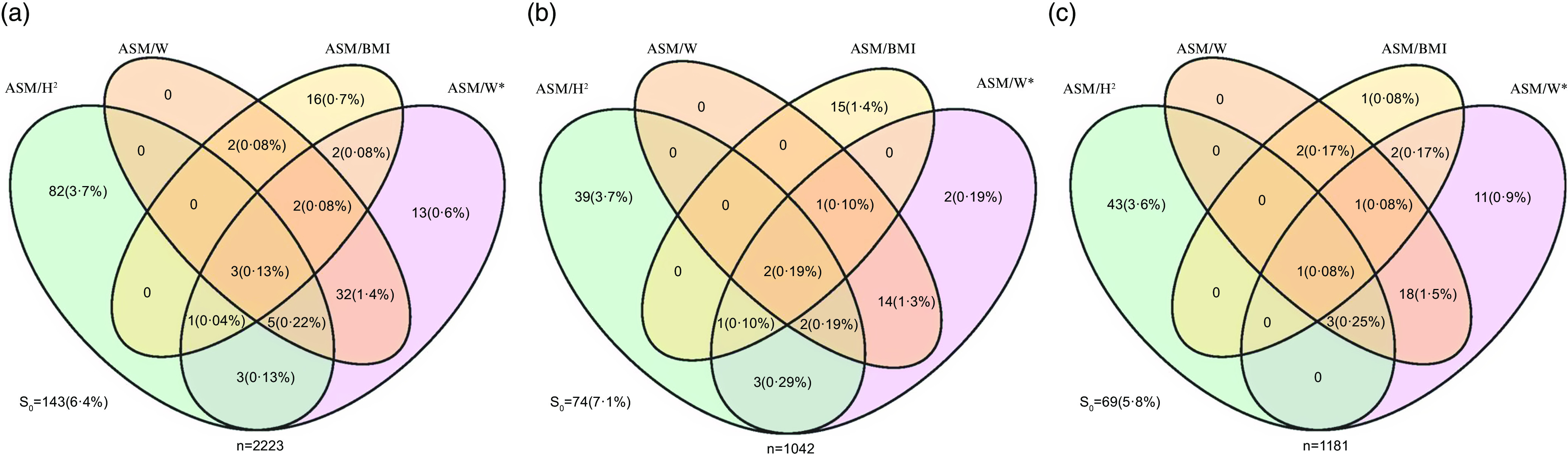
Venn diagram showed the overlap of prevalence of LMM defined by each assessment methods. The diagrams show the number and frequency of LMM defined by only one up to all five methods. Data are shown for all (a), male (b) and female (c) participants. ASM, appendicular skeletal mass; ASM/H^2^, ASM/height^2^; ASM/W, ASM/weight. The cut-off values for LMM were defined by ASM/H^2^ < 7·0 kg/m^2^ for males or < 5·7 kg/m^2^ for females; ASM/W < 29·0 % for males or < 22·9 % for females; and ASM/BMI < 0·789 for males or < 0·512 for females. ASM/W* = ASM/W × 100 %, beyond 2 sd below the sex-specific mean for healthy young adults in this study and the cut-off values was < 29·5 % for males or < 23·5 % for females; S_0_, satisfied ASM/H^2^ or ASM/W or ASM/BMI or ASM/W*. LMM, low muscle mass.

The prevalence of LMM evaluated by the five measurement methods, and their overlap in all, male and female subjects were shown in Fig. 2(a)–(c). For the whole population (*n* 2223), 143 (6·4 %), 91 (4·1 %), 44 (2·0 %), 24 (1·1 %) and 59 (2·7 %) of the participants were determined as LMM when using S_0_, ASM/H^2^, ASM/W, ASM/BMI or ASM/W*, respectively. The highest overlap rate was observed in ASM/W with ASM/W* in all (42, 1·9 %), males (19, 1·8 %) and females (23, 1·9 %); the lowest was for ASM/H^2^ with ASM/BMI (4 (0·38 %), 3 (0·27 %) and 1 (0·08 %), respectively).

**Fig. 3.**
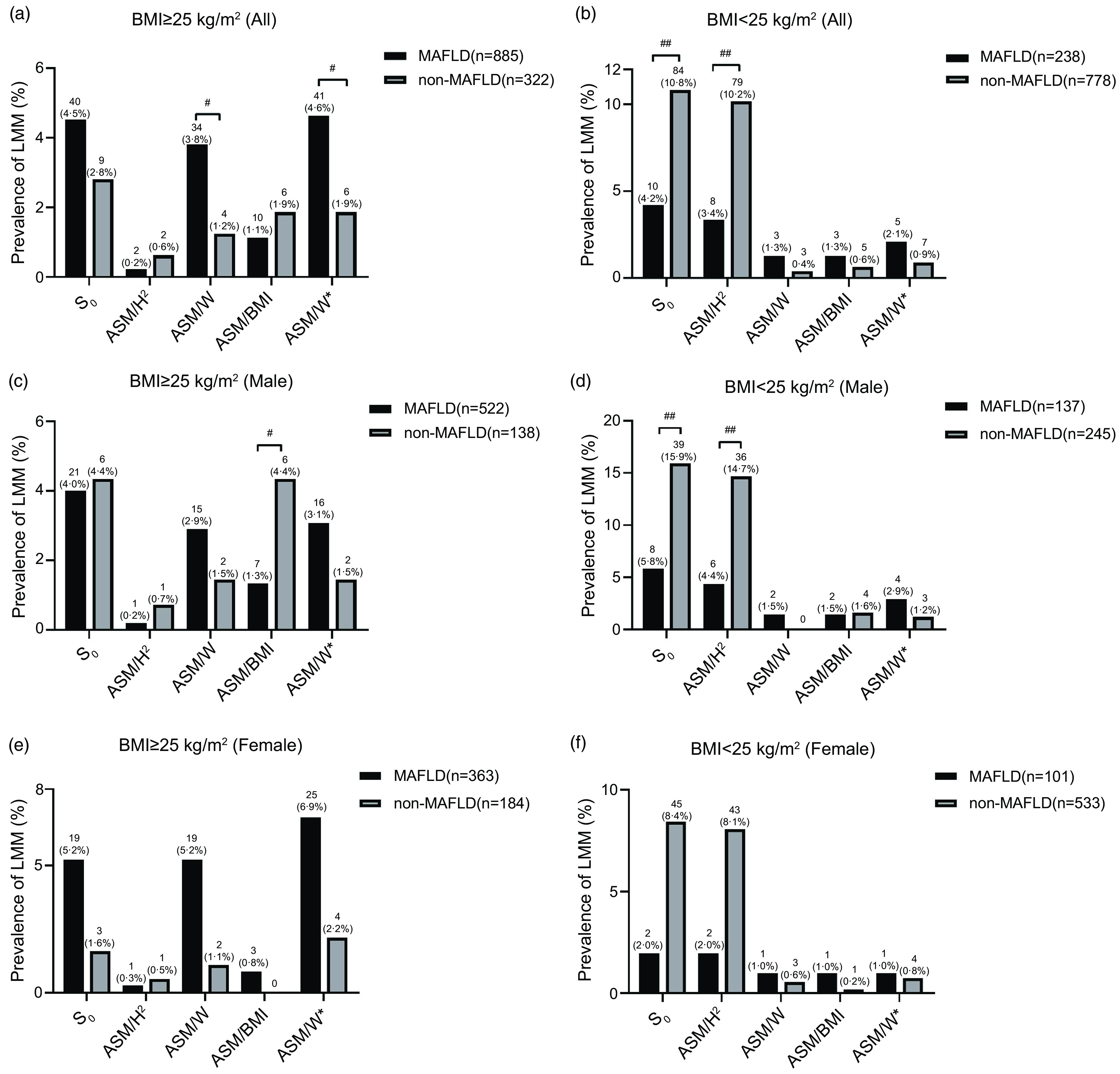
Comparison of prevalence of LMM in MAFLD and non-MAFLD classified by BMI in all (a), (d), male (b), (e) and female (c), (f) subjects with five assessment methods. MAFLD, metabolic-associated fatty liver disease; ASM, appendicular skeletal mass; ASM/H^2^, ASM/height^2^; ASM/W, ASM/weight; ASM/BMI, ASM/BMI; ASM/W* = ASM/W × 100 %, beyond 2 sd below the sex-specific mean for healthy young adults in this study; S_0_, satisfied ASM/H^2^ or ASM/W or ASM/BMI or ASM/W*. ^#^ *P* < 0·05, ^##^ *P* < 0·01, ^###^ *P* < 0·001.

Furthermore, MAFLD and non-MAFLD subjects in male and female were stratified by obesity status. Obesity was defined as BMI ≥ 25 kg/m^2^ using the BMI criteria for Asian populations recommended by the WHO^(35)^. The prevalence of LMM in those without and with obesity varied by assessment methods of LMM. Assessed by ASM/W, more subjects were identified as LMM in MAFLD than non-MAFLD in all, male and female subjects with obesity (3·4 % *v*. 1·2 %, *P* < 0·05; 2·1 % *v*. 1·5 %; 5·2 % *v*. 1·1 %; Fig. 3(a), (c), (e)), while the result was significant just for all subjects. In contrast, the results were the opposite when using ASM/H^2^ in subjects without obesity (3·4 % *v*. 10·2 %, *P* < 0·01; 4·4 % *v*. 14·7 %, *P* < 0·01; 2·0 % *v*. 8·1 %, Fig. 3(b), (d), (f)), and the results were significant for all and male subjects.

### Association of appendicular skeletal muscle index with metabolic-associated fatty liver disease prevalence

**Fig. 4.**
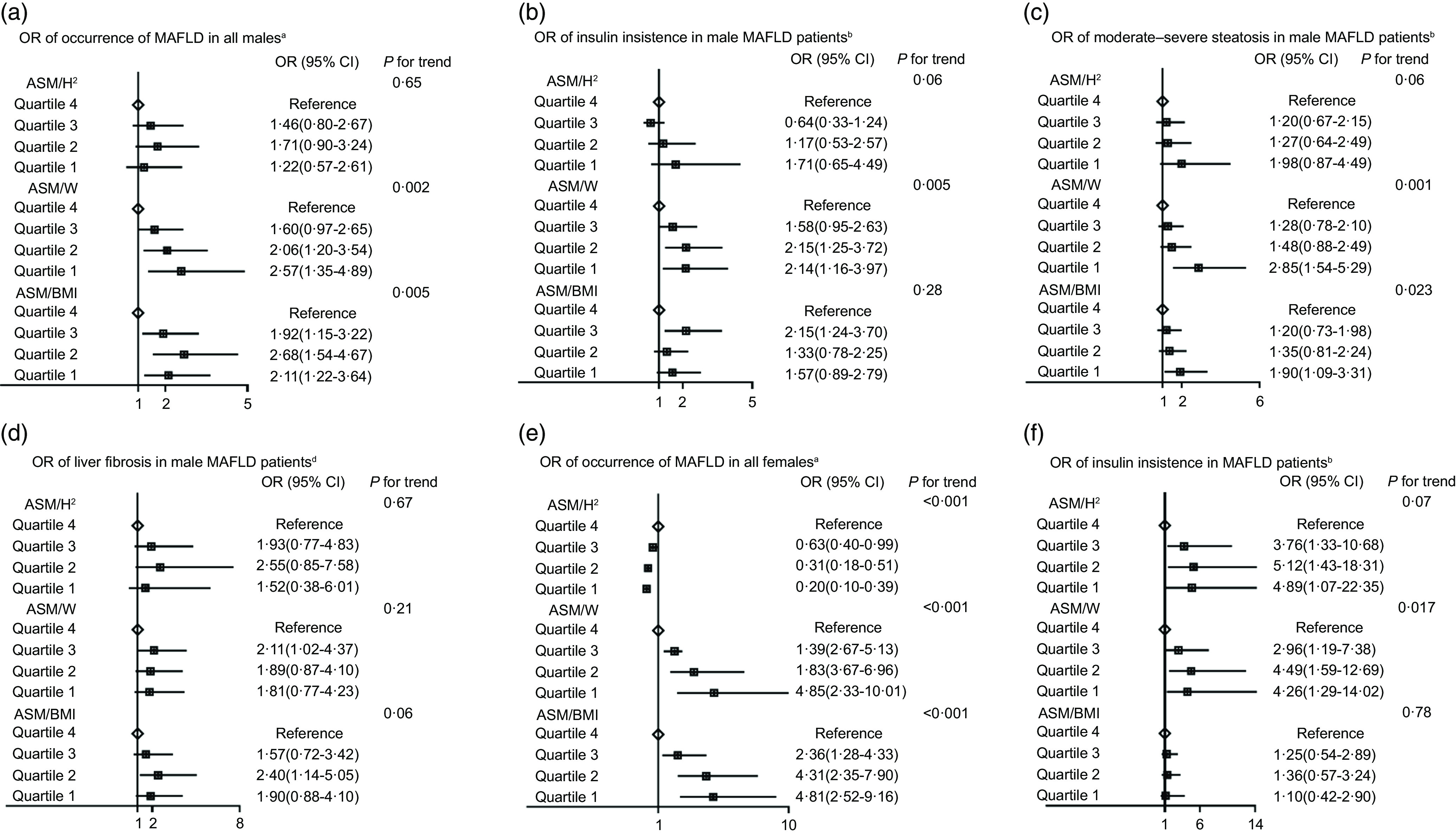
The risks of MAFLD in all male subjects (a) and risks of insulin resistance, moderate–severe steatosis and fibrosis in male MAFLD patients (b), (c) and (d). The risks of MAFLD in all female subjects (e) and risks of insulin resistance in female MAFLD patients (f). MAFLD, metabolic-associated fatty liver disease; ASM, appendicular skeletal mass; ASM/H^2^, ASM/height^2^; ASM/W, ASM/weight. Insulin resistance is defined as homeostasis model assessment of insulin resistance ≥ 2·5. Moderate–severe steatosis is defined as liver fat content ≥ 10 %. Liver fibrosis is defined as liver stiffness measurement ≥ 7·1kPa. ^a^The multivariate logistic regression model was adjusted for age, waist circumference, TAG, diabetes and uric acid. ^b^The multivariate logistic regression model was adjusted for age, BMI and diabetes. ^c^The multivariate logistic regression model was adjusted for age, BMI, waist circumference, TAG and diabetes. ^d^The multivariate logistic regression model was adjusted for age and BMI.

Univariate logistic regression suggested that the OR (95 % CI) for incident MAFLD with ASM/H^2^, ASM/W or ASM/BMI were 2·11 (95 % CI 1·85, 2·40), 0·71 (95 % CI 0·67, 0·75) and 0·08 (95 % CI 0·02, 0·32), respectively, for male, 3·25 (95 % CI 2·75, 3·85), 0·62 (95 % CI 0·58, 0·66) and 0·15 (95 % CI 0·01, 0·12) for female, respectively (Table 2). The participants were subgrouped into ASM/H^2^, ASM/W or ASM/BMI quartiles stratified by sex to be analysed for multivariate logistic regression, which were represented as quartile 1 (Q1), quartile 2 (Q2), quartile 3 (Q3) and quartile 4 (Q4) from lowest to highest (the 25 %, 50 % and 75 % quartiles for all male subjects: ASM/H^2^ (kg/m^2^): 8·1, 8·9, 9·9; ASM/W (%): 33·4, 34·7, 36·6; ASM/BMI: 0·95, 1·02, 1·09; for all female subjects: ASM/H^2^ (kg/m^2^): 6·5, 7·1, 7·7; ASM/W (%): 26·6, 28·5, 30·8; ASM/BMI: 0·67, 0·72, 0·79). The Q4 in each group was set as a reference. After adjusting for potential confounding factors including age, waist circumference, TAG, diabetes and uric acid, in both sexes, the subjects with the lowest quartile (Q1) of ASM/W or ASM/BMI had higher risk ratios for MAFLD compared with the highest (Q4) (OR (95 % CI) in male: 2·57 (1·35, 4·89), 2·11(1·22, 3·64); in female: 4·85 (2·33, 10·01), 4·81 (2·52, 9·16), all *P* < 0·05), respectively (Fig. 4(a) and (e)). However, the subjects with the lowest quartile (Q1) of ASM/H^2^ had lowest risk ratios for MAFLD (0·20 (0·10, 0·39), *P* < 0·05), merely in female (Fig. 4(e)). The predictive value of ASM/H^2^ for MAFLD disappeared in multivariate logistic regression in male.

**Table 2.** OR for risk of MAFLD, insulin resistance, moderate–severe steatosis and liver fibrosis with ASM/H^2^, ASM/W and ASM/BMI in males and females

Groups	OR (95 % CI)					
	ASM/H^2^		ASM/W		ASM/BMI	
	OR	95 % CI	OR	95 % CI	OR	95 % CI
Risk of MAFLD						
All males (*n* 1042)	2·11	1·85, 2·40***	0·71	0·67, 0·75***	0·08	0·02, 0·32***
All females (*n* 1181)	3·25	2·75, 3·85***	0·62	0·58, 0·66***	0·15	0·01, 0·12***
Risk of IR						
Males (*n* 659)†	1·46	1·25, 1·70***	0·79	0·73, 0·85***	0·09	0·02, 0·49**
Females (*n* 464)†	1·04	0·75, 1·46^NS^	0·84	0·75, 0·95**	0·13	0·01, 4·33^NS^
Risk of moderate–severe steatosis						
Males (*n* 659)‡	1·15	1·01, 1·32*	0·90	0·84, 0·97**	0·41	0·07, 2·23^NS^
Females (*n* 464)‡	1·31	0·76, 2·24^NS^	0·97	0·82, 1·14^NS^	1·11	0·81, 1·53^NS^
Risk of liver fibrosis						
Males (*n* 386)§	1·36	1·11, 1·68**	0·82	0·72, 0·93**	0·05	0·01, 0·68*
Females (*n* 103)§	0·69	0·29, 1·65^NS^	0·82	0·64, 1·06^NS^	0·79	0·49, 1·25^NS^

MAFLD, metabolic-associated fatty liver disease; IR, insulin resistance; ASM, appendicular skeletal mass; ASM/H^2^, ASM/height^2^; ASM/W, ASM/weight.

Insulin resistance is defined as homeostasis model assessment of insulin resistance ≥ 2·5. Moderate–severe steatosis is defined as liver fat content ≥ 10 %. Liver fibrosis is defined as liver stiffness measurement ≥ 7·1kPa.

*P < 0·05, **P < 0·01, ***P < 0·001.

† The number of male and female MAFLD patients with HOMA-IR were 659 and 464, respectively.

‡ The number of male and female MAFLD patients undergoing MRI-PDFF were 659 and 464, respectively.

§ The number of male and female MAFLD patients undergoing 2D-SWE were 386 and 103, respectively.

### Association of appendicular skeletal muscle index with insulin resistance, steatosis and fibrosis

Univariate logistic regression analysis was conducted in male and female MAFLD patients, and the results showed that the ASM/H^2^, ASM/W and ASM/BMI could be predictors for IR, moderate–severe steatosis and fibrosis for both sexes (Table 2). Therefore, male and female MAFLD patients were divided into ASM/H^2^, ASM/W or ASM/BMI quartiles (the 25 %, 50 % and 75 % quartiles for male MAFLD: ASM/H^2^ (kg/m^2^): 8·7, 9·4, 10·3; ASM/W (%): 32·4, 33·7, 35·3; ASM/BMI: 0·93, 1·00, 1·05; for female MAFLD: ASM/H^2^ (kg/m^2^): 7·0, 7·6, 8·2; ASM/W (%): 24·9, 26·4, 28·0; ASM/BMI: 0·62, 0·67, 0·71), and multivariable logistic regression analysis was furthermore performed.

In multivariate-adjusted model, the MAFLD patients with lower quartiles of ASM/W had the higher risk OR for IR, both in male and female (2·14 (1·16–3·97), 4·26 (1·29–14·02) for Q4 *v*. Q1, both *P* < 0·05). While the significant OR were not observed in MAFLD patients in the groups of ASM/H^2^ and ASM/BMI (Fig. 4(b) and (f)). For moderate–severe steatosis, there were significant dose-dependent associations between decreased ASM/W as well as ASM/BMI and moderate–severe steatosis (*P* for trend = 0·001 and 0·023) in male MAFLD patients. There was no such association in female MAFLD patients (online Supplementary Fig. 1(a)). In this study, a total of 318 male and 103 female MAFLD patients underwent 2D-SWE to evaluate liver stiffness. There was no significant difference in age between all MAFLD and MAFLD patients with 2D-SWE in both sexes (online Supplementary Table 1 and 2). In the model adjusted by age and BMI, there were no significant OR for fibrosis both in male and female MAFLD patients with three assessments methods (Fig. 4(d) and online Supplementary Fig. 1(b)).

Associations of ASM/W with steatosis and fibrosis by hepatic histology were further analysed. A total of fifty-eight male and twenty-one female MAFLD were available for liver biopsy reports. There were no differences in age between all and biopsy-proven MAFLD patients in male and female (online Supplementary Table 1 and 2). In male, the ASM/W was significantly higher in mild (S1, 34·8 ± 1·7 %) than moderate (S2, 33·5 ± 1·6 %) and severe (S3, 32·9 ± 2·7 %) steatosis (*P* = 0·014, *P* = 0·015, respectively), while no difference was observed between F0 and F1–F3 (online Supplementary Fig. 2(a) and (c)). For female, the ASM/W showed no differences in the subgroups which classified by steatosis and fibrosis (All *P* > 0·05, Supplementary Fig. 2(b) and (d)).

### Comparison in predictive values of appendicular skeletal muscle index for metabolic-associated fatty liver disease, insulin resistance, steatosis and fibrosis

**Fig. 5.**
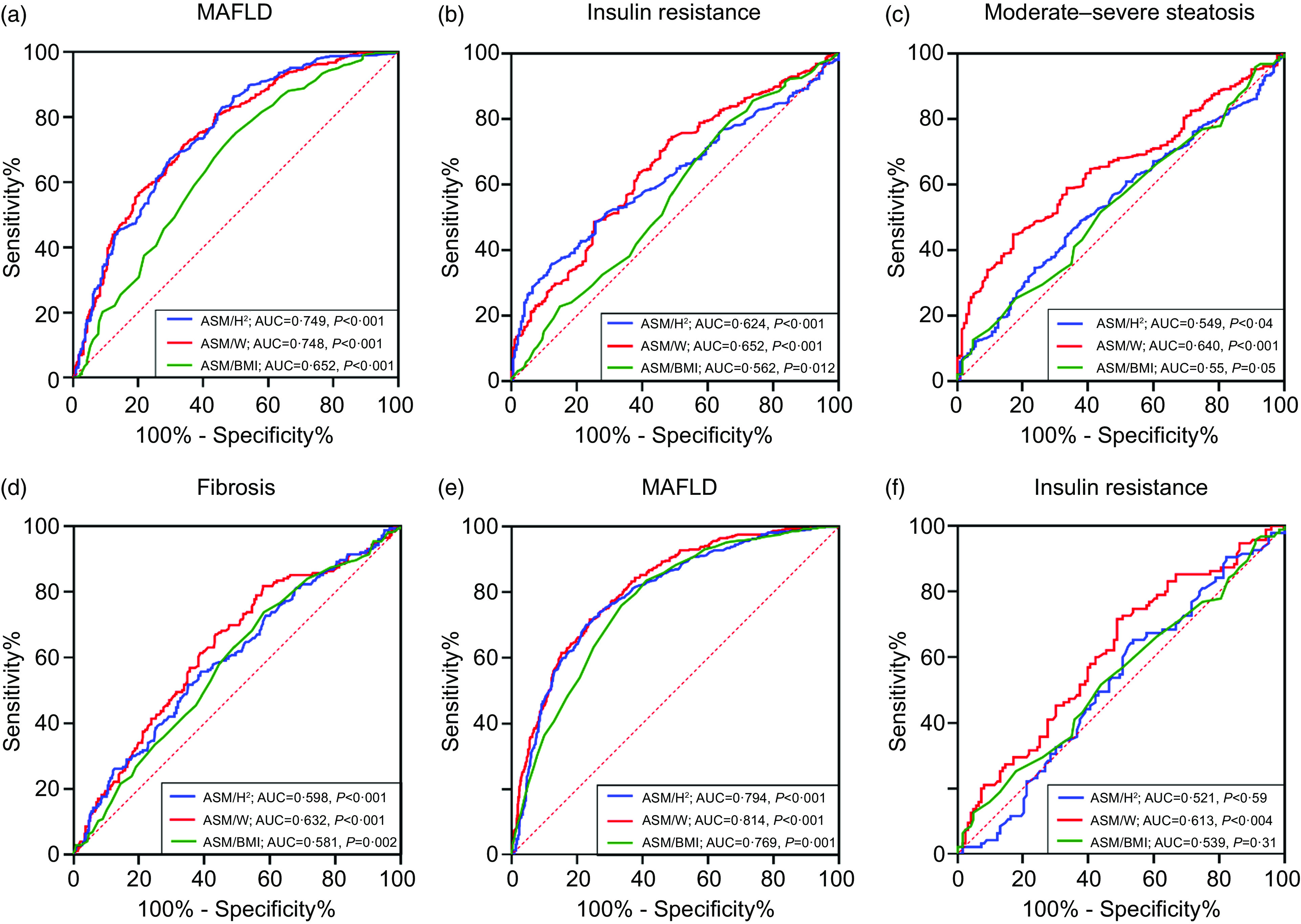
Receiver operator characteristic (ROC) curve predicting MAFLD for male (a) and insulin resistance, moderate–severe steatosis and fibrosis in male MAFLD patients (b), (c) and (d) with three assessment methods. ROC curve predicting for MAFLD in all female subjects (e) and insulin resistance in female MAFLD patients (f). MAFLD, metabolic associated fatty liver disease; ASM, appendicular skeletal mass; ASM/H^2^, ASM/height^2^; ASM/W, ASM/weight.

ASM/H^2^, ASM/W and ASM/BMI were used to construct receiver operator characteristic curves for predicting MAFLD, IR, moderate–severe steatosis and fibrosis. For males and females, the areas under the receiver operator characteristic curve for predicting MAFLD were 0·749 and 0·794 for ASM/H^2^, 0·748 and 0·814 for ASM/W, and 0·652 and 0·769 for ASM/BMI (all *P* < 0·001), respectively (Fig. 5(a) and (e)). Regarding IR, ASM/H^2^, ASM/W and ASM/BMI attained an AUC of 0·624, 0·652 and 0·562 (all *P* < 0·05) in male MAFLD, while significant AUC was merely observed in ASM/W (0·613, *P* = 0·004), not in ASM/H^2^ and ASM/BMI in female MAFLD (Fig. 5(b) and (f)). ASM/W showed the highest AUC for predicting moderate–severe steatosis (0·640, *P* < 0·001) and liver fibrosis (0·632, *P* < 0·001) in male MAFLD (Fig. 5(c) and (d)), while significant AUC were not achieved in female MAFLD (online Supplementary Fig. 3a and 3b).

## Discussion

The association between low skeletal muscle mass and MAFLD has been verified by previous studies, while the data in non-elderly population are limited. This is the first study to explore the prevalence of LMM in MAFLD with different ASMI estimation methods (ASM/H^2^, ASM/W and ASM/BMI) in non-elderly Asian population, further investigating the associations of which with IR, steatosis grades and hepatic fibrosis.

Focusing on non-elderly patients, we found that ASMI decreased in MAFLD subjects when determined with ASM/W and ASM/BMI, whereas it increased with ASM/H^2^. First proposed in 1998, ASM/H^2^ is positively related to BMI and thus has the limitation of underestimating sarcopenia in subjects with higher BMI. Therefore, ASM/W was suggested in 2002 as an alternative method. In 2014, ASM/BMI was introduced and gradually became widely used^(36)^. Our results suggested that absolute muscle mass (ASM/H^2^) increased, while relative muscle mass (ASM/W and ASM/BMI) decreased in MAFLD. Another study reported similar results^(19)^. This may be because absolute muscle mass increases with increasing weight in MAFLD, as ASM/H^2^ is adjusted by height^2^, a relatively stable index not affected by lifestyle compared with weight. The lower ASM/W and ASM/BMI in MAFLD might be explained by that there is less muscle gain than weight gain in MAFLD. Interestingly, decrease of ASM/W or ASM/BMI was observed in male and female MAFLD, while handgrip strength showed no difference between MAFLD and non-MAFLD groups. It is considered that non-elderly MAFLD patients just had a loss of muscle mass without decrease of muscle function.

The Venn diagram showed that the prevalence of LMM varied widely with different assessments, so overlaps were very low. A recent study also reported that only 0·29 % (4/1343) of the participants satisfied the criteria for LMM according to both weight- and height^2^-adjusted ASMI at the same time^(16)^. Previous studies have explored the discrepancies among different assessment methods for LMM^(16,19,37)^. NAFLD is associated with a lower risk of sarcopenia when using the height-adjusted SMI. In contrast, it showed the opposite result when using the weight-adjusted SMI^(19)^. A study reported weakness of ASM/H^2^ is that subjects with a greater BMI are less likely to be identified as having sarcopenia^(37)^. On the one hand, the low overlaps and difference of the prevalence of LMM among different methods may be due to the limitation of BIA, because the measurement of muscle mass may be overestimated or underestimated, which can be influenced by hydrated status and illness^(38,39)^. On the other hand, this may be explained by that the influence of body fat mass is not taken into account in ASM/H^2(40)^. Therefore, ASM/H^2^ might not be suitable for evaluating the prevalence of sarcopenia in Asian MAFLD patients with a BMI ≥ 25 kg/m^2^. Another study also demonstrated that the prevalence of sarcopenia (defined by weight-adjusted ASMI) was significantly higher in the MAFLD group than in the control group (8·8 % *v*. 1·3 %, *P* < 0·001). However, the opposite result was obtained with the height^2^-adjusted ASMI (MAFLD: 0·8 %; control: 2·0 %, *P* = 0·055)^(16)^. Similar results were found in this study that the prevalence of LMM in MAFLD was significantly higher than non-MAFLD in all subjects with obesity (3·4 % *v*. 1·2 %, *P* < 0·05), while the result was the opposite with ASM/H^2^ (3·4 % *v*. 10·2 %, *P* < 0·01). Different from the above studies, non-elderly MAFLD patients were divided by BMI and sex to further compare the effects among various diagnostic methods in our study, and the results indicated that the prevalence of LMM in non-elderly subjects with obesity (BMI ≥ 25 kg/m^2^) might be underestimated when using ASM/H^2^. Nonetheless, the prevalence may be underestimated when using ASM/W in non-elderly MAFLD patients without obesity (BMI < 25 kg/m^2^). Therefore, ASM/W is suitable for evaluation of LMM in MAFLD patients with obesity, but ASM/H^2^ is better in MAFLD patients without obesity.

Published studies have reported the association between sarcopenia and MAFLD, with most concluding that loss of skeletal muscle mass increased the risk of MAFLD^(12,41)^. A prospective observational study including 452 subjects (median age > 50) observed that individuals with lower muscle mass adjusted by weight exhibited an increased risk of MAFLD^(12)^. Another retrospective study involving 5989 subjects (mean age 53·2 ± 9·4) suggested that a low ASM/W quartile was significantly associated with an increased prevalence of MAFLD (OR, 1·28; 95 % CI, 1·21, 1·37; *P* < 0·001)^(41)^. These two studies evaluated the risk of MAFLD in older populations. The similar conclusion was drawn in the non-elderly population in this study, whereby the subjects with lower ASM/W or ASM/BMI had a higher risk of MAFLD. Different from those studies, we also analysed the association between ASM/H^2^ and MAFLD and found the opposite result in females, compared with ASM/W or ASM/BMI. The discrepancy was also reported by Peng and his colleagues^(19)^. It raised our attention on how we choose assessment methods of ASMI when the association between ASM and MAFLD is explored. A recent meta-analysis reported nineteen studies exploring the relationship between SMI and MAFLD, of which ASM/W, ASM/BMI and ASM/H^2^ were used in 16, 3 and 3 studies, respectively^(42)^. ASM/W is used more commonly than the other two and showed LMM is related to MAFLD^(12,21,41,43,44)^.

Existing studies have reported a relationship of skeletal muscle mass and sarcopenia with IR^(8,45)^. Skeletal muscle mass (adjusted by body weight) was found to be inversely associated with IR in a large population from the Third National Health and Nutrition Examination Survey^(8)^. Another study concluded that obese men with sarcopenia (defined by ASM/W) exhibited a significantly higher IR risk than those without obese^(45)^. A similar relationship was observed in our study. A lower ASMI adjusted by weight predicting a higher risk of IR both in male and female MAFLD, which might be explained by the underlying relationship between sarcopenia and IR. Skeletal muscle is a major target organ for insulin in which glucose metabolism is mediated by insulin. Subjects with obesity present increased fatty acid infiltration of muscle or myosteatosis, which reduces muscle dysfunction^(46)^. In MAFLD, loss of skeletal muscle and muscle dysfunction exacerbate IR^(47)^. Furthermore, it has been reported that in young and middle-aged individuals, sarcopenia is associated with inflammation (higher serum CRP levels), which is recognised as a central mediator of obesity-associated IR^(48)^. On the other hand, IR participates in the occurrence of sarcopenia. IR might inhibit protein synthesis in skeletal muscle through the mammalian target of rapamycin complex 1 (mTORC1) or ribosomal protein S6 kinase beta-1 (S6 K1) pathway^(49,50)^. In general, the mechanism of the interactions of IR and sarcopenia is not fully understood and needs further exploration.

LMM is significantly associated with the severity of steatosis in MAFLD^(14,18,41,51)^. A retrospective study that enrolled 5989 subjects indicated that sarcopenia (diagnosed by ASM/W) independently increased the severity of MAFLD evaluated by ultrasonography^(41)^. A recent prospective study enrolling 3014 participants who were followed up for 2 years reported that a higher hepatic steatosis index and fatty liver index increased the risk of LMM (defined by ASM/BMI)^(18)^. Two prospective studies (including 309 and 225 subjects) reported that sarcopenia (defined by ASM/W) was associated with an increased risk of non-alcoholic steatohepatitis based on the gold standard (histological evidence)^(14,51)^. In the present study, we firstly analysed the association between ASMI and the severity of steatosis assessed by the liver fat content fraction based on MRI-PDFF. In accordance with previous studies, a lower ASM/W and ASM/BMI increased the risk of moderate–severe steatosis in males with MAFLD after adjusting cofounders. ASM/W decreased with the liver steatosis grade increased in the histological analysis in male MAFLD, which may provide more solid evidence about their relationships. However, the relationship disappeared for ASM/H^2^. The results from previous and our studies suggested that ASM/W was a better choice to analyse the relationship between ASM and metabolic diseases or factors, such as MAFLD and IR. Interestingly, the association was not observed in females, which may be explained by differences in sex hormones and fat distribution. Indeed, oestrogen protects against MAFLD in females owing to its antisteatotic, antioxidant and antifibrogenic effects on the liver^(43)^. Moreover, body fat tends to accumulate relatively more in the hips and thighs in females, instead of the upper body, including abdominal visceral fat compared with males^(45)^.

Associations between ASM and fibrosis in MAFLD patients has also been reported^(13,14,51,52)^. Two cross-sectional studies concluded that sarcopenia (defined by ASM/BMI and ASM/W) increased the risk of liver fibrosis evaluated by the NAFLD fibrosis score, FIB-4 and Forns index in 2761 and 4188 subjects with MAFLD from NHANES^(13,52)^. Another two prospective studies enrolled 309 and 225 subjects and found sarcopenia (determined by ASM/W) to be a risk factor for significant fibrosis evaluated by biopsy^(14,51)^. In our study, none of three ASMI can be a significant predictor for fibrosis in multivariate analysis, and ASM/W showed no difference in MAFLD with or without fibrosis based on histological data. This may be caused by two reasons. Firstly, the baseline liver stiffness measurement measured by 2D-SWE was low, suggesting MAFLD had no or mild fibrosis in our study. Second, the number of patients undergoing 2D-SWE was small. Therefore, further investigation with a large sample on the relationship between ASM and fibrosis is necessary.

The strength of this study is that we firstly explored the association of skeletal muscle mass with the prevalence and severity of MAFLD in non-elderly population with different methods evaluating ASMI. Several studies have reported the relationship between MAFLD and ASMI, which was adjusted by height^2^ and weight in cohorts with a higher average age^(16,19)^. This study demonstrated that the subjects with lower ASM/W had a higher risk of MAFLD in younger population (mean age 37·4 ± 10·6 years) and increased the risk of IR and severer liver steatosis in male. The result suggested that decrease in ASM/W also need concern in non-elderly people. Second, MRI-PDFF was utilised to evaluate the liver steatosis in this study, which has been proven to have excellent diagnostic value for liver fat content and histologic steatosis in MAFLD patients^(53)^. However, there are some limitations in this study. First, paraments of body composition was obtained by BIA, which estimated the skeletal muscle mass by the equation, instead of dual-energy X-ray absorptiometry (DXA), the gold standard for the measurement of muscle mass. Although BIA showed good correction with DXA, the BIA algorithms were developed by the manufacturer in specific population and adjustment equations remain required in clinical application^(38)^. Even if the use of BIA for the evaluation of ASM is considered acceptable in different documents (only if DXA is not available), most of the researchers suggest caution in clinical practice and even more in scientific research^(54,55)^. Over or under-estimation of ASM is frequent with BIA compared with DXA, and there is the necessity to adopt adjustment equations that need to be ethnicity-/age-/sex-/disease-specific. However, the equation used in the current study developed by Yamada was validated a good correlation with DXA independent of age in Asian (Japanese)^(28)^, which provided a higher accuracy for ASM measurement for the population in this study. DXA requires radiation exposure and is expensive. In comparison, BIA enables patients to avoid radiation exposure and is affordable, portable and easy to use. Besides, this study demonstrated that the choice of method to screen LMM is based on obesity status of patients. ASM/W has the advantage on evaluation of LMM in non-elderly MAFLD patients with obesity, while ASM/H^2^ is better in non-elderly MAFLD patients without obesity. And ASM/W is an independent predictor for IR and moderate-to-severe steatosis in male non-elderly MAFLD; therefore, it is superior to ASM/H^2^ and ASM/BMI. Second, it was a cross-sectional study without furthermore follow-up of ASMI and severity of MAFLD. The longitudinal investigation of the association of the change in ASMI with the change in hepatic steatosis and IR is needed. Third, this is a single-centre study, so the multicentre studies based on a large sample are warranted to provide more powerful evidence^(10)^. Last, because this study included only Chinese subjects, the conclusions might not be generalisable to other ethnicities.

In conclusion, the prevalence of LMM differs with the population stratified by sex and BMI. Based on population classified by BMI, ASM/W is suitable for people with obesity, while ASM/H^2^ is better for those without obesity. ASM/W is superior to ASM/H^2^ and ASM/BMI in predicting the degree of MAFLD. A lower ASM/W is related to IR and moderate–severe steatosis in non-elderly males with MAFLD but not in females.

## Supplementary material

For supplementary material accompanying this paper visit https://doi.org/10.1017/S0007114523000399. click here to view supplementary material
