# Supplementary material for: Impact of skeletal muscle mass evaluating methods on severity of metabolic associated fatty liver disease in non-elderly adults
Source: Br J Nutr. 2023 Mar 10;130(8):1373–84. doi: 10.1017/S0007114523000399 (PMC10511683; doi:10.1017/S0007114523000399)
Supplement: Supplementary file 1 [file S0007114523000399sup001.docx]

**Supplementary materials**

**Impact of skeletal muscle mass evaluating methods on severity of**

**metabolic associated fatty liver disease in nonelderly adults**

**British Journal of Nutrition**

Ting Zhou^1#^, Junzhao Ye^1#^, Yansong Lin^1^, Wei Wang^2^, Shiting Feng^3^, Shuyu Zhuo^4*^, Bihui Zhong^1*^

**Affiliations**

1. Department of Gastroenterology, The First Affiliated Hospital, Sun Yat-sen University, No. 58 Zhongshan II Road, Yuexiu District, Guangzhou, 510080 China.

2. Department of Medical Ultrasonics, Institute of Diagnostic and Interventional Ultrasound, The First Affiliated Hospital, Sun Yat-sen University, No. 58 Zhongshan II Road, Yuexiu District, Guangzhou, Guangdong, 510080 China.

3. Department of Radiology, The First Affiliated Hospital, Sun Yat-sen University, No. 58 Zhongshan II Road, Yuexiu District, Guangzhou, Guangdong, 510080 China.

4. Department of Nutrition, The First Affiliated Hospital, Sun Yat-sen University, No. 58 Zhongshan II Road, Yuexiu District, Guangzhou, Guangdong, 510080 China.

*Corresponding author:

Shuyu Zhuo, MD, PhD

Department of Nutrition, The First Affiliated Hospital, Sun Yat-sen University, Guangzhou, No. 58 Zhongshan II Road, Yuexiu District, Guangzhou, 510080 China

Phone: (020) 87755766

Email: zhuoshy@mail.sysu.edu.cn

Bihui Zhong, MD, PhD

Department of Gastroenterology, The First Affiliated Hospital, Sun Yat-sen University, No. 58 Zhongshan II Road, Yuexiu District, Guangzhou, 510080 China

Phone: (020) 87755766

Email: zhongbh@mail.sysu.edu.cn

**Supplementary table 1. Comparison of the baseline characteristics between all MAFLD and MAFLD undergoing 2D-SWE as well as MAFLD proven by biopsy in males.**

| **Characteristics** | **All MAFLD (n=659)** | **MAFLD undergoing 2D-SWE (n=386)** | ***P*^a^** | **MAFLD proven by biopsy (n=58)** | ***P* ^b^** |
| --- | --- | --- | --- | --- | --- |
| **Demographics** |  |  |  |  |  |
| **Age(years)** | 37.1±10.5 | 37.5±10.0 | 0.63 | 35.5±11.6 | 0.14 |
| **Anthropometry** |  |  |  |  |  |
| **Weight (kg)** | 83.0±18.0 | 81.3±15.7 | 0.07 | 79.2±15.9 | 0.07 |
| **BMI (kg/m^2^)** | 28.9±5.8 | 27.6±3.3 | 0.08 | 27.1±4.5 | 0.09 |
| **Waist circumference (cm)** | 97.1±11.2 | 96.2±8.7 | 0.06 | 94.8±10.7 | 0.08 |
| **Waist-hip ratio** | 0.87±0.07 | 0.88±0.25 | 0.29 | 0.87±0.27 | 0.33 |
| **Liver biochemistry** |  |  |  |  |  |
| **Alanine aminotransferase (U/L)** | 42(26, 69) | 49(32, 80) | ＜0.001 | 77(32, 121) | ＜0.001 |
| **Aspartate aminotransferase (U/L)** | 30(23, 42) | 33(25, 47) | 0.001 | 43(27, 60) | ＜0.001 |
| **γ-glutamyl transpeptidase (U/L)** | 39(28, 65) | 48(32, 77) | ＜0.001 | 62(43, 127) | ＜0.001 |
| **Alkaline phosphatase (U/L)** | 75(66, 88) | 77(66, 91) | 0.28 | 81(74, 96) | 0.005 |
| **Metabolic characteristics** |  |  |  |  |  |
| **Total cholesterol (mmol/L)** | 5.3±1.1 | 5.3±1.1 | 0.66 | 5.2±0.9 | 0.32 |
| **Triglyceride (mmol/L)** | 1.8(1.2, 2.5) | 1.7(1.2, 2.3) | 0.91 | 1.9(1.4, 2.4) | 0.33 |
| **HDL-C (mmol/L)** | 1.1±0.6 | 1.1±0.4 | 0.83 | 1.1±0.2 | 0.49 |
| **LDL-C (mmol/L)** | 3.3±0.8 | 3.4±0.8 | 0.97 | 3.3±0.7 | 0.38 |
| **Fast serum glucose (mmol/L)** | 5.5±1.6 | 5.4±1.5 | 0.11 | 5.1±1.0 | 0.08 |
| **HOMA-IR** | 2.4(1.6, 3.7) | 2.3(1.7, 3.5) | 0.90 | 2.5(2.0, 4.6) | 0.15 |
| **Uric acid (μmol/L)** | 444.3±107.7 | 459±107 | 0.05 | 458±95 | 0.37 |
| **Liver stiffness measurement** |  |  |  |  |  |
| **Liver stiffness (kPa)** | 6.6±2.4 | 6.6±2.4 | 1.00 | 6.7±2.0 | 0.64 |
| **Liver steatosis** |  |  |  |  |  |
| **Liver fat content (%)** | 12(8, 20) | 13(8, 20) | 0.66 | 18(8, 24) | 0.05 |
| **Body composition** |  |  |  |  |  |
| **ASM (kg)** | 28.2±5.7 | 28.0±4.8 | 0.27 | 26.7±4.8 | 0.05 |
| **ASM/H^2^ (kg/m^2^)** | 9.5±1.6 | 9.4±1.2 | 0.18 | 9.2±1.3 | 0.15 |
| **ASM/W (%)** | 33.6±2.9 | 34.2±2.9 | 0.11 | 34.1±1.9 | 0.54 |
| **ASM/BMI(m^2^)** | 0.99±0.14 | 1.02±0.11 | 0.13 | 0.99±0.10 | 0.71 |

Continuous variables are reported as mean ± standard deviation (SD) or median (IQR).

MAFLD, metabolic associated fatty liver disease; 2D-SWE, two-dimensional shear wave elastography; BMI, body mass index; HDL-C, high-density lipoprotein cholesterol; LDL-C, low-density lipoprotein cholesterol; HOMA-IR, homeostatic model assessment of insulin resistance; ASM, appendicular skeletal mass; ASM/H^2^, ASM/height^2^; ASM/W, ASM/weight; ASM/BMI, ASM/body mass index.

*P* ^a^, The *P* value between all MAFLD and MAFLD undergoing 2D-SWE patients; *P* ^b^, The *P* value between all MAFLD and MAFLD proven by biopsy patients.

**Supplementary table 2. Comparison of the baseline characteristics between all MAFLD and MAFLD undergoing 2D-SWE as well as MAFLD proven by biopsy in females.**

| **Characteristics** | **All MAFLD (n=464)** | **MAFLD undergoing 2D-SWE (n=103)** | ***P*^a^** | **MAFLD proven by biopsy (n=21)** | ***P* ^b^** |
| --- | --- | --- | --- | --- | --- |
| **Demographics** |  |  |  |  |  |
| **Age(years)** | 38.9±10.8 | 41.1±9.0 | 0.12 | 41.0±13.0 | 0.45 |
| **45Anthropometry** |  |  |  |  |  |
| **Weight (kg)** | 73.4±12.7 | 65.2±9.8 | ＜0.001 | 64.7±8.9 | 0.006 |
| **BMI (kg/m^2^)** | 28.9±4.2 | 25.8±3.3 | ＜0.001 | 26.2±2.9 | 0.009 |
| **Waist circumference (cm)** | 94.0±10.1 | 87.6±8.6 | ＜0.001 | 88.1±7.0 | 0.021 |
| **Waist-hip ratio** | 0.88±0.11 | 0.86±0.15 | 0.11 | 0.83±0.24 | 0.10 |
| **Liver biochemistry** |  |  |  |  |  |
| **Alanine aminotransferase (U/L)** | 24(15, 38) | 28(18, 56) | 0.022 | 47(30, 99) | ＜0.001 |
| **Aspartate aminotransferase (U/L)** | 22(18, 31) | 25(20, 40) | 0.048 | 41(31, 74) | ＜0.001 |
| **γ-glutamyl transpeptidase (U/L)** | 27(22, 38) | 41(19, 69) | 0.10 | 54(41, 93) | ＜0.001 |
| **Alkaline phosphatase (U/L)** | 70(60, 80) | 69(61, 85) | 0.78 | 84(64, 103) | 0.016 |
| **Metabolic characteristics** |  |  |  |  |  |
| **Total cholesterol (mmol/L)** | 5.6±1.5 | 5.2±0.9 | 0.013 | 5.1±0.9 | 0.12 |
| **Triglyceride (mmol/L)** | 1.5(1.1, 2.2) | 1.5(1.1, 1.9) | 0.45 | 1.5(1.1, 1.7) | 0.57 |
| **HDL-C (mmol/L)** | 1.3±0.4 | 1.2±0.2 | 0.035 | 1.3±0.3 | 0.67 |
| **LDL-C (mmol/L)** | 3.5±1.2 | 3.2±0.7 | 0.063 | 3.1±0.7 | 0.23 |
| **Fast serum glucose (mmol/L)** | 5.6±1.6 | 5.5±1.8 | 0.79 | 5.±1.1 | 0.72 |
| **HOMA-IR** | 2.1(1.5, 3.5) | 2.1(1.4, 3.6) | 0.77 | 2.2(1.2, 3.5) | 0.99 |
| **Uric acid (μmol/L)** | 346±93 | 359±90 | 0.25 | 499±143 | 0.036 |
| **Liver stiffness measurement** |  |  |  |  |  |
| **Liver stiffness (kPa)** | 6.1±3.3 | 6.1±3.. | 1.00 | 8.1±7.4 | 0.08 |
| **Liver steatosis** |  |  |  |  |  |
| **Liver fat content (%)** | 12(8, 21) | 11(8, 20) | 0.55 | 18(8, 30) | 0.27 |
| **Body composition** |  |  |  |  |  |
| **ASM (kg)** | 19.5±2.8 | 17.7±3.0 | <0.001 | 17.9±2.4 | 0.028 |
| **ASM/H^2^ (kg/m^2^)** | 7.7±0.9 | 7.1±0.7 | <0.001 | 7.2±0.7 | 0.038 |
| **ASM/W (%)** | 26.6±2.5 | 27.7±2.1 | 0.001 | 27.8±2.0 | 0.10 |
| **ASM/BMI(m^2^)** | 0.69±0.23 | 0.70±0.07 | 0.77 | 0.68±0.07 | 0.77 |

Continuous variables are reported as mean ± standard deviation (SD) or median (IQR).

MAFLD, metabolic associated fatty liver disease; 2D-SWE, two-dimensional shear wave elastography; BMI, body mass index; HDL-C, high-density lipoprotein cholesterol; LDL-C, low-density lipoprotein cholesterol; HOMA-IR, homeostatic model assessment of insulin resistance; ASM, appendicular skeletal mass; ASM/H^2^, ASM/height^2^; ASM/W, ASM/weight; ASM/BMI, ASM/body mass index.

*P* ^a^, The *P* value between all MAFLD and MAFLD undergoing 2D-SWE patients; *P* ^b^, The *P* value between all MAFLD and MAFLD proven by biopsy patients.


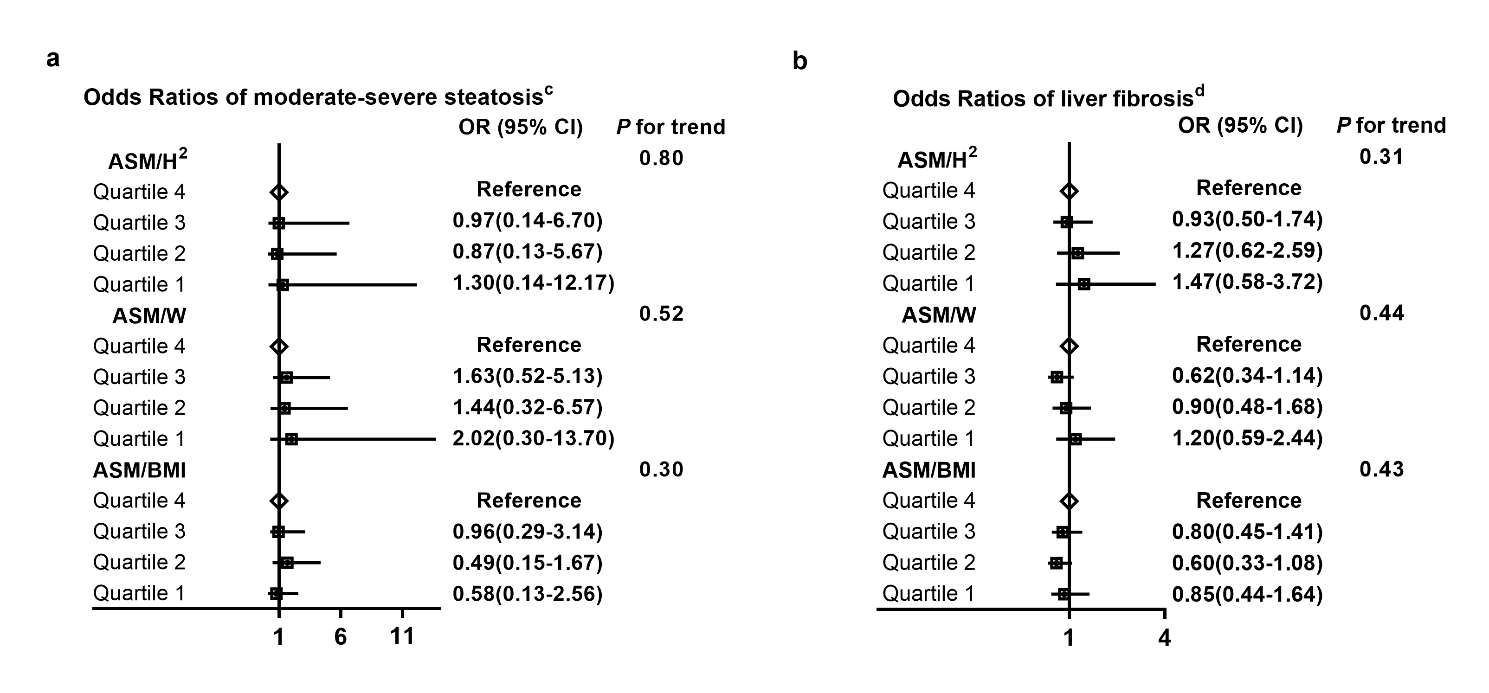


**Supplementary fig 1.** The risks of moderate-severe steatosis and fibrosis in female MAFLD patients.

MAFLD, metabolic associated fatty liver disease; ASM, appendicular skeletal mass; ASM/H^2^, ASM/height^2^; ASM/W, ASM/weight; ASM/BMI, ASM/body mass index. Moderate-severe steatosis is defined as liver fat content ≥10%. Liver fibrosis is defined as liver stiffness measurement ≥7.1kPa.

^c^ The multivariate logistic regression model was adjusted for age, body mass index, waist circumference, triglyceride and diabetes.

^d^ The multivariate logistic regression model was adjusted for age and body mass index.

**Supplementary fig 2.** Comparison of ASM/W among steatosis ang fibrosis grade by liver biopsy in male (a, c) and female MAFLD groups (b, d), respectively

MAFLD, metabolic associated fatty liver disease; ASM, appendicular skeletal mass; ASM/W, ASM/weight. **P*<0.05, NS, not significant.

*


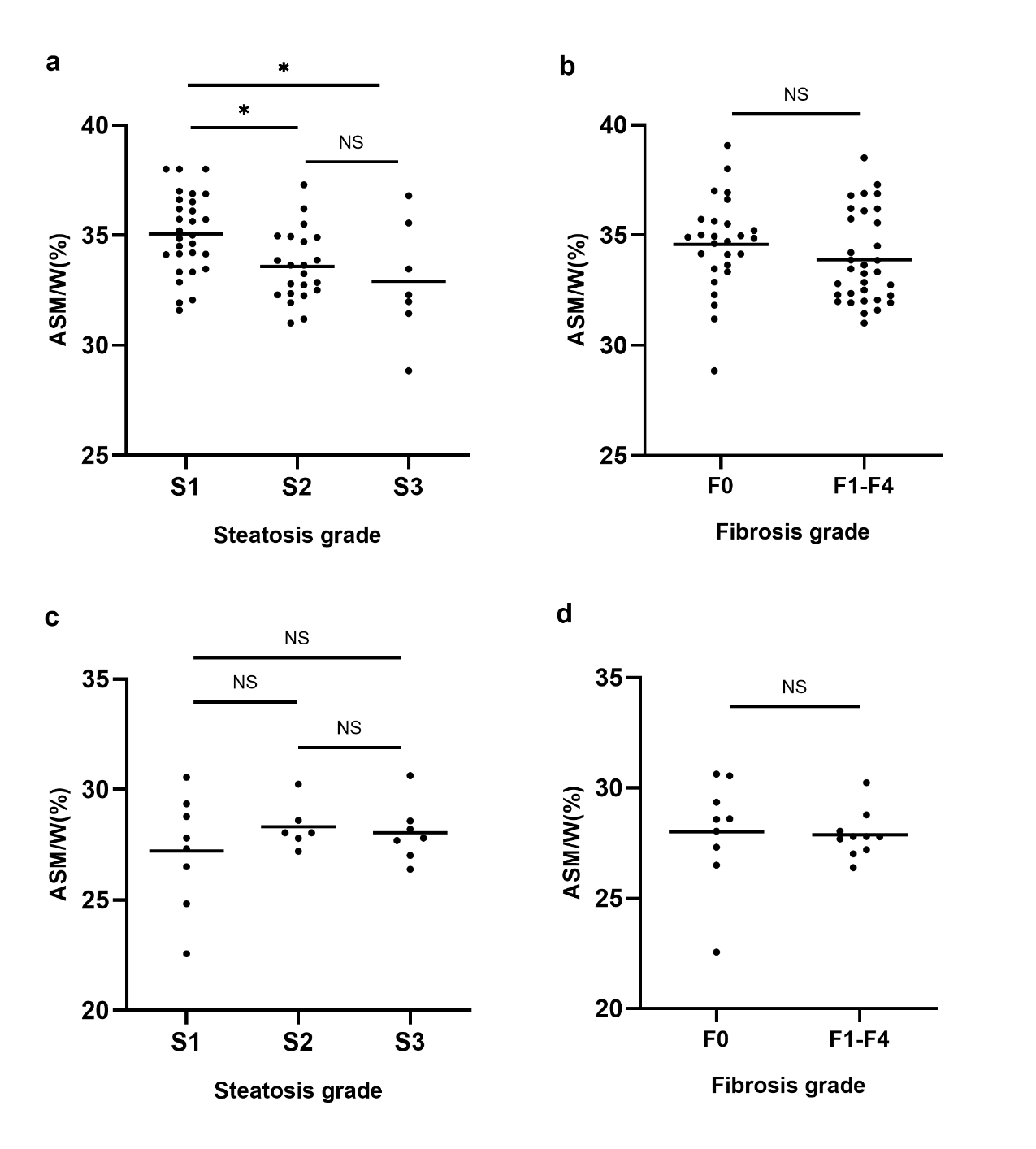


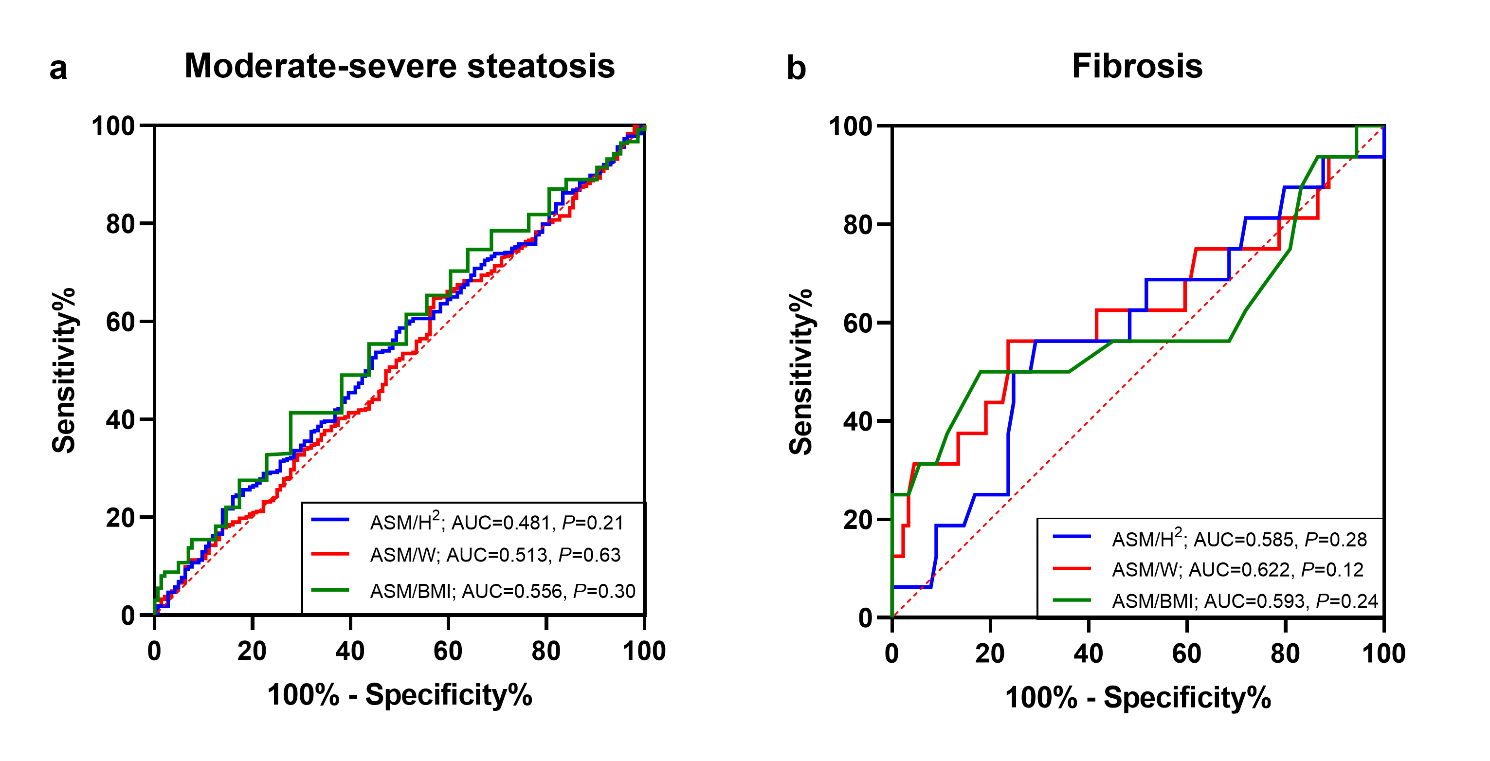


**Supplementary fig 3**. Receiver operator characteristic (ROC) curve predicting moderate-severe steatosis (a) and fibrosis (b) for female MAFLD patients with 3 assessment methods.

MAFLD, metabolic associated fatty liver disease; ASM, appendicular skeletal mass; ASM/H^2^, ASM/height^2^; ASM/W, ASM/weight; ASM/BMI, ASM/body mass index; AUC, area under curve.
